# Machine learning algorithm–based predictive model of late-life loneliness level in long-term care residents

**DOI:** 10.1093/geroni/igaf122.4310

**Published:** 2025-12-31

**Authors:** Mo Yi, Zhiwen Wang, Anna Beeber

**Affiliations:** Peking University, Beijing, Beijing, China (People’s Republic); Peking University, Beijing, Beijing, China (People’s Republic); Johns Hopkins University, Baltimore, Maryland, United States

## Abstract

**Background and Purpose:**

Late-life loneliness affects nearly one-third of older adults in long-term care facilities (LCFs) and is strongly associated with adverse health outcomes, posing a serious threat to global healthy aging. This study aimed to construct and validate predictive models using five advanced machine learning algorithms to identify residents at risk of loneliness and to rank key contributing factors.

**Methods:**

Data were collected from 583 residents across six LCFs in China who had stayed for at least three months. Loneliness was defined as a UCLA-8 score ≥16. Sixteen predictors covering demographic, physical, psychological, and social domains were selected via LASSO approach to train five machine learning algorithms with cross-validation. Model performance was evaluated by the area under the curve (AUC), accuracy, recall, specificity, precision, and Cohen’s kappa.

**Results:**

The ElasticNet algorithm model achieved the best overall performance, with an AUC of 0.737. At the 0.5 probability threshold, accuracy was 70.6%, specificity 95.3%, precision 70.8%, recall 71.5%, and kappa 0.404. The confusion matrix showed strong ability to identify residents without loneliness but limited sensitivity for detecting loneliness. Feature ranking revealed the top eight predictors as depression, anxiety, instrumental activities of daily living, vision status, multimorbidity, cognitive orientation deficit, language ability, and mobility. Psychological symptoms and functional impairments consistently contributed the greatest weight.

**Conclusion:**

Findings suggest its potential use for routine screening and may assist care staff in early identification and targeted interventions for loneliness and the promising use of machine learning for care of older adults in LCFs.

